# Supplementary material for: Delay of punishment highlights differential vulnerability to developing addiction-like behavior toward sweet food
Source: Transl Psychiatry. 2024 Mar 20;14:155. doi: 10.1038/s41398-024-02863-6 (PMC10954751; doi:10.1038/s41398-024-02863-6)
Supplement: Supplementary file 1 — Supplementary Figures [file 41398_2024_2863_MOESM1_ESM.pptx]

## Slide 1
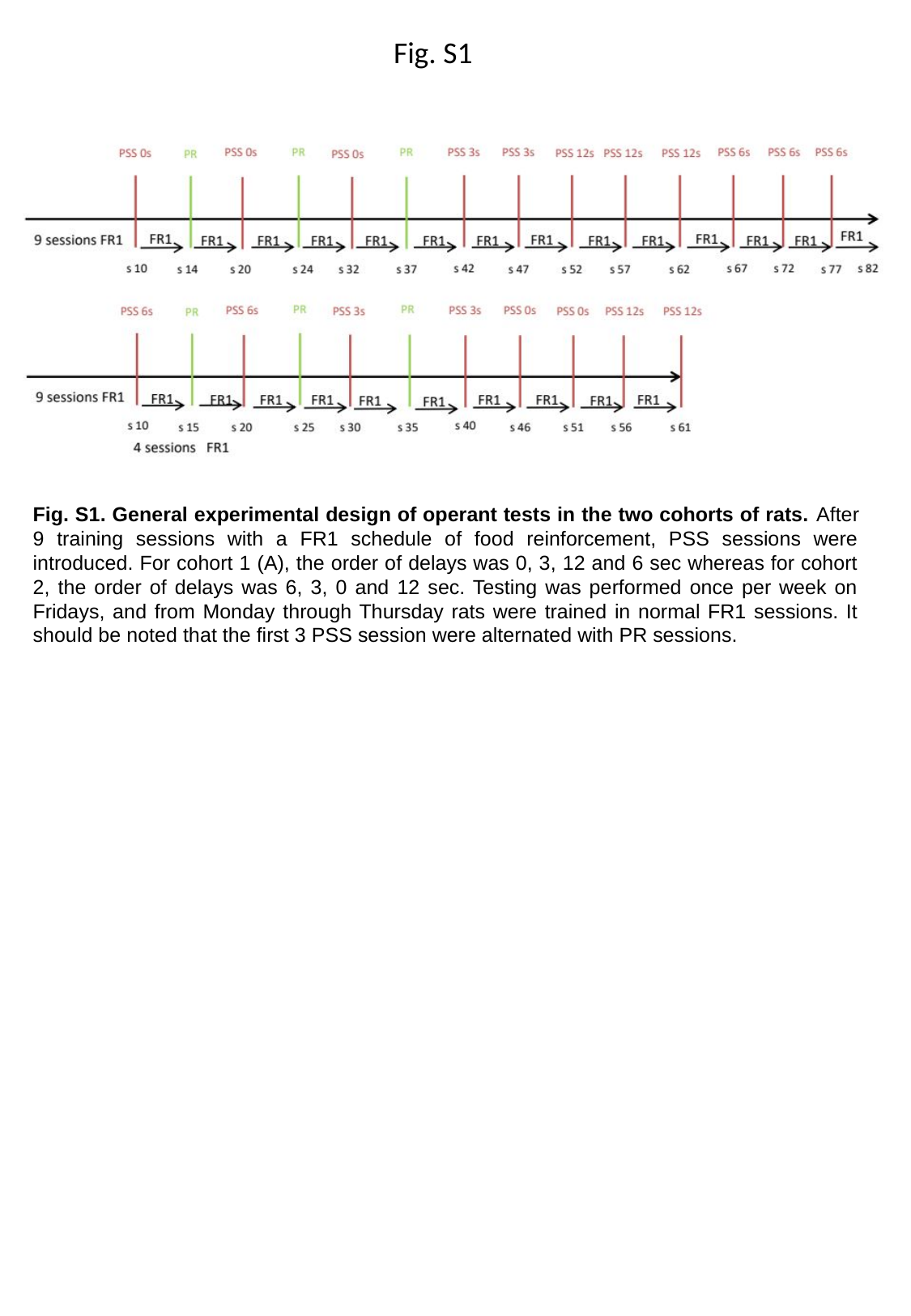

Fig. S1
Fig. S1. General experimental design of operant tests in the two cohorts of rats. After 9 training sessions with a FR1 schedule of food reinforcement, PSS sessions were introduced. For cohort 1 (A), the order of delays was 0, 3, 12 and 6 sec whereas for cohort 2, the order of delays was 6, 3, 0 and 12 sec. Testing was performed once per week on Fridays, and from Monday through Thursday rats were trained in normal FR1 sessions. It should be noted that the first 3 PSS session were alternated with PR sessions.

## Slide 2
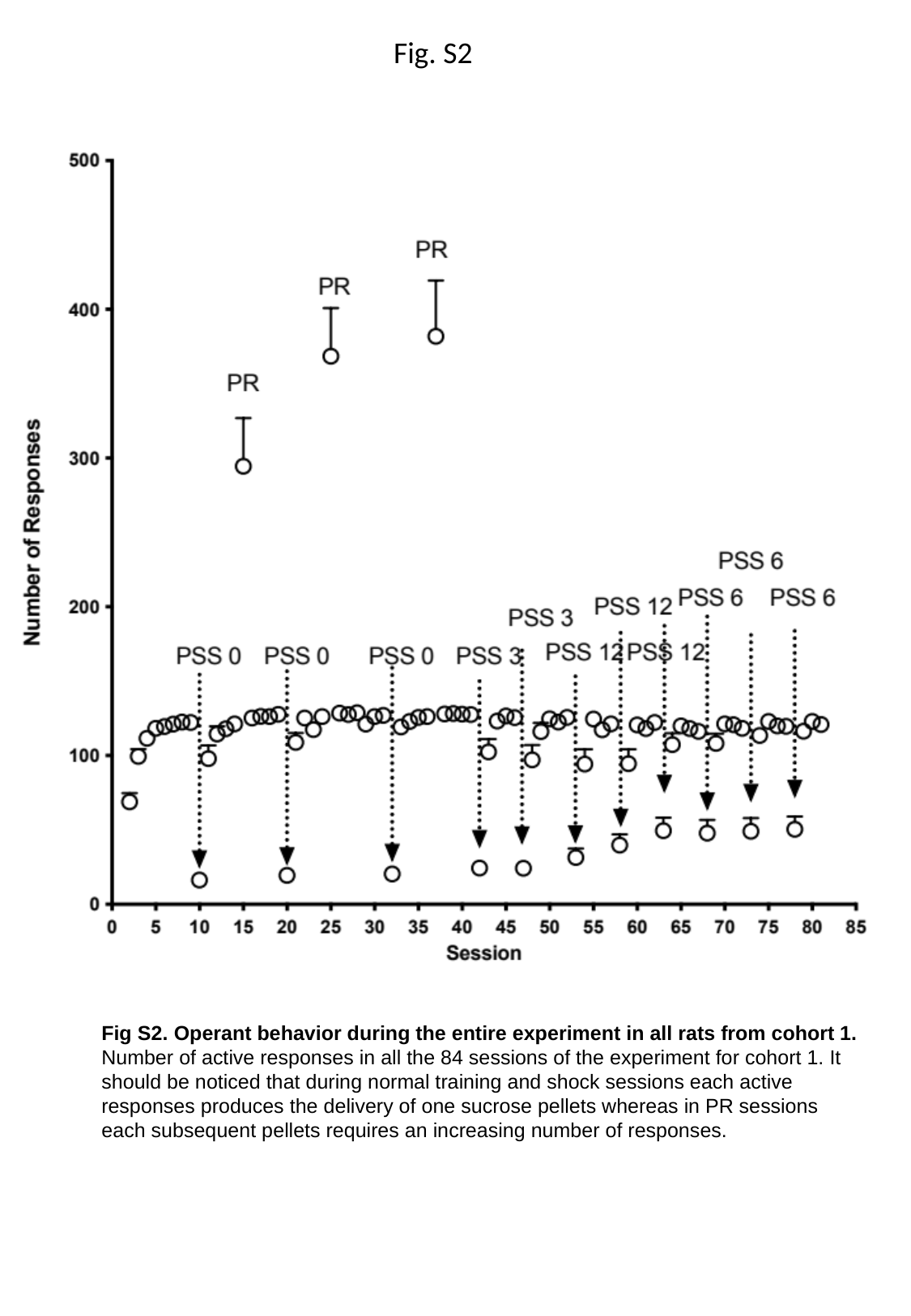

Fig. S2
Fig S2. Operant behavior during the entire experiment in all rats from cohort 1. Number of active responses in all the 84 sessions of the experiment for cohort 1. It should be noticed that during normal training and shock sessions each active responses produces the delivery of one sucrose pellets whereas in PR sessions each subsequent pellets requires an increasing number of responses.

## Slide 3
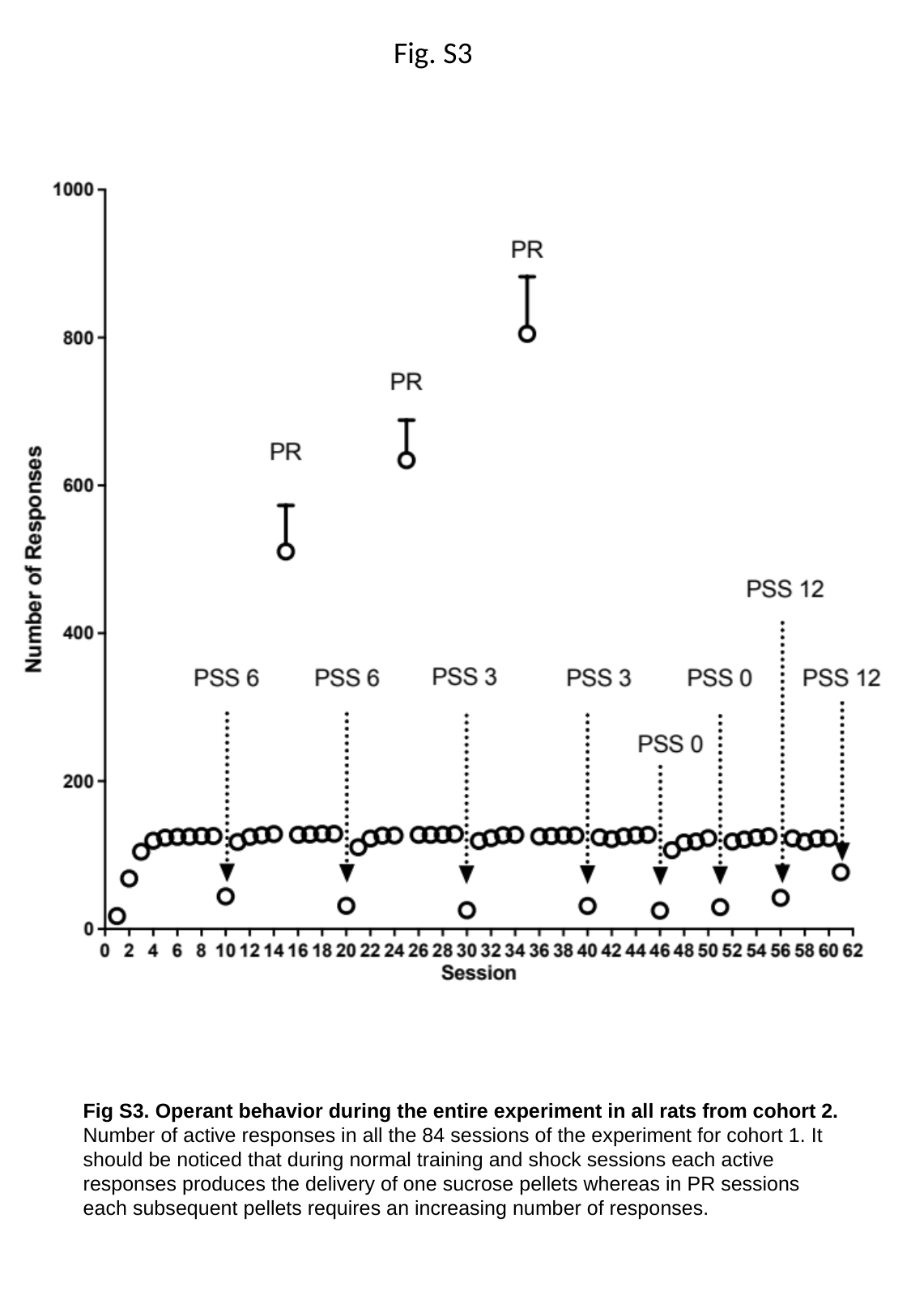

Fig. S3
Fig S3. Operant behavior during the entire experiment in all rats from cohort 2. Number of active responses in all the 84 sessions of the experiment for cohort 1. It should be noticed that during normal training and shock sessions each active responses produces the delivery of one sucrose pellets whereas in PR sessions each subsequent pellets requires an increasing number of responses.

## Slide 4
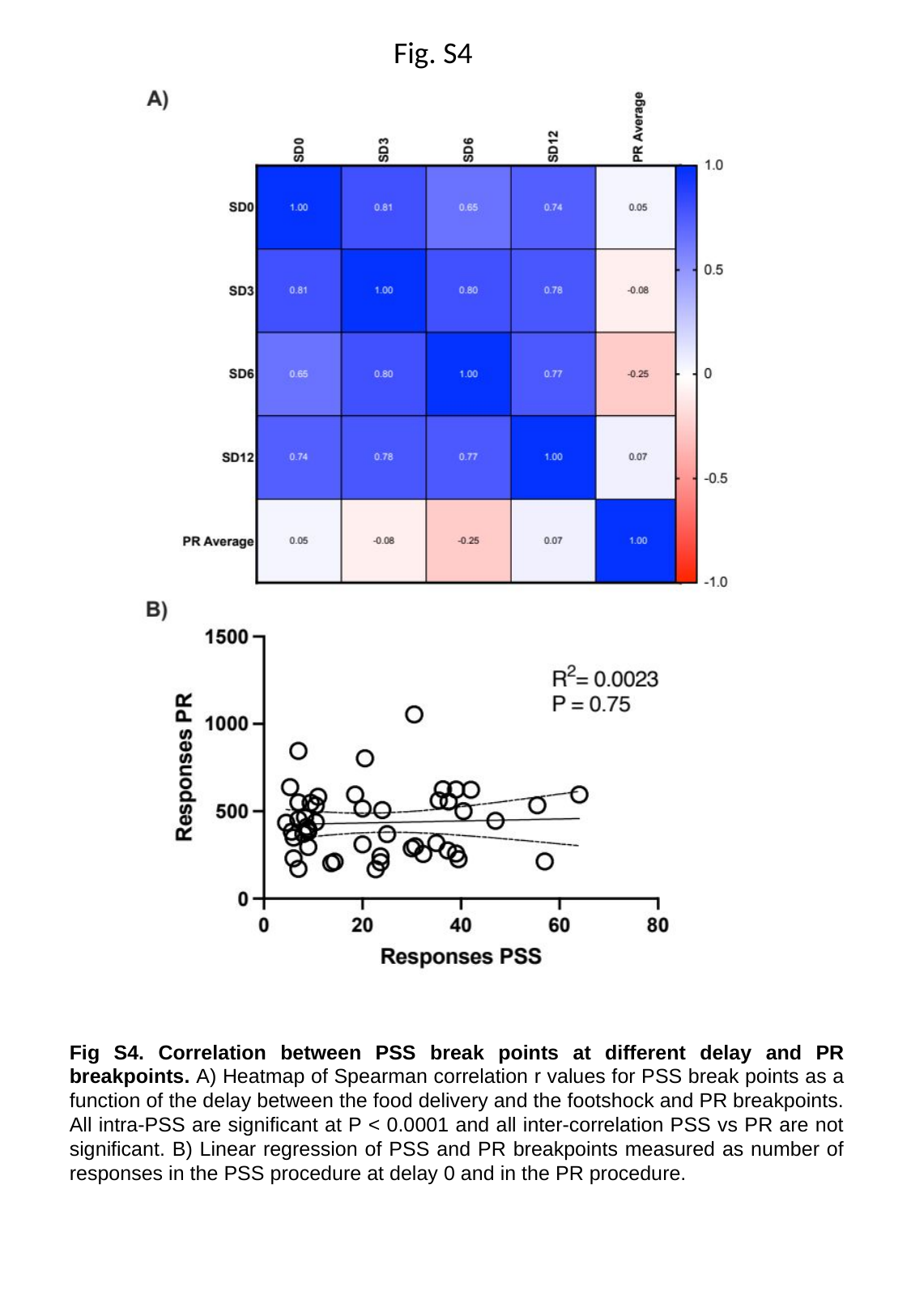

Fig. S4
Fig S4. Correlation between PSS break points at different delay and PR breakpoints. A) Heatmap of Spearman correlation r values for PSS break points as a function of the delay between the food delivery and the footshock and PR breakpoints. All intra-PSS are significant at P < 0.0001 and all inter-correlation PSS vs PR are not significant. B) Linear regression of PSS and PR breakpoints measured as number of responses in the PSS procedure at delay 0 and in the PR procedure.

## Slide 5
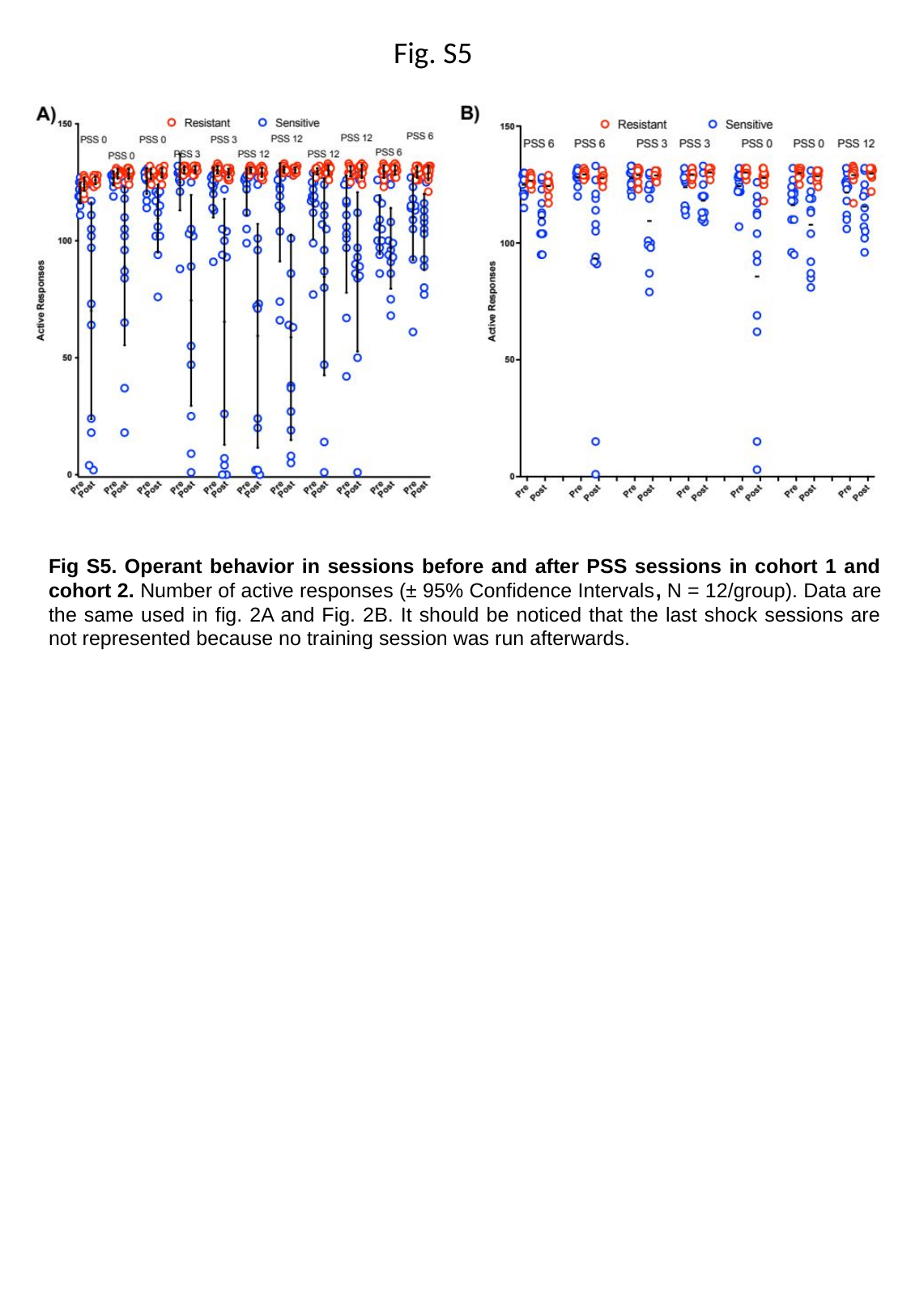

Fig. S5
Fig S5. Operant behavior in sessions before and after PSS sessions in cohort 1 and cohort 2. Number of active responses (± 95% Confidence Intervals, N = 12/group). Data are the same used in fig. 2A and Fig. 2B. It should be noticed that the last shock sessions are not represented because no training session was run afterwards.

## Slide 6
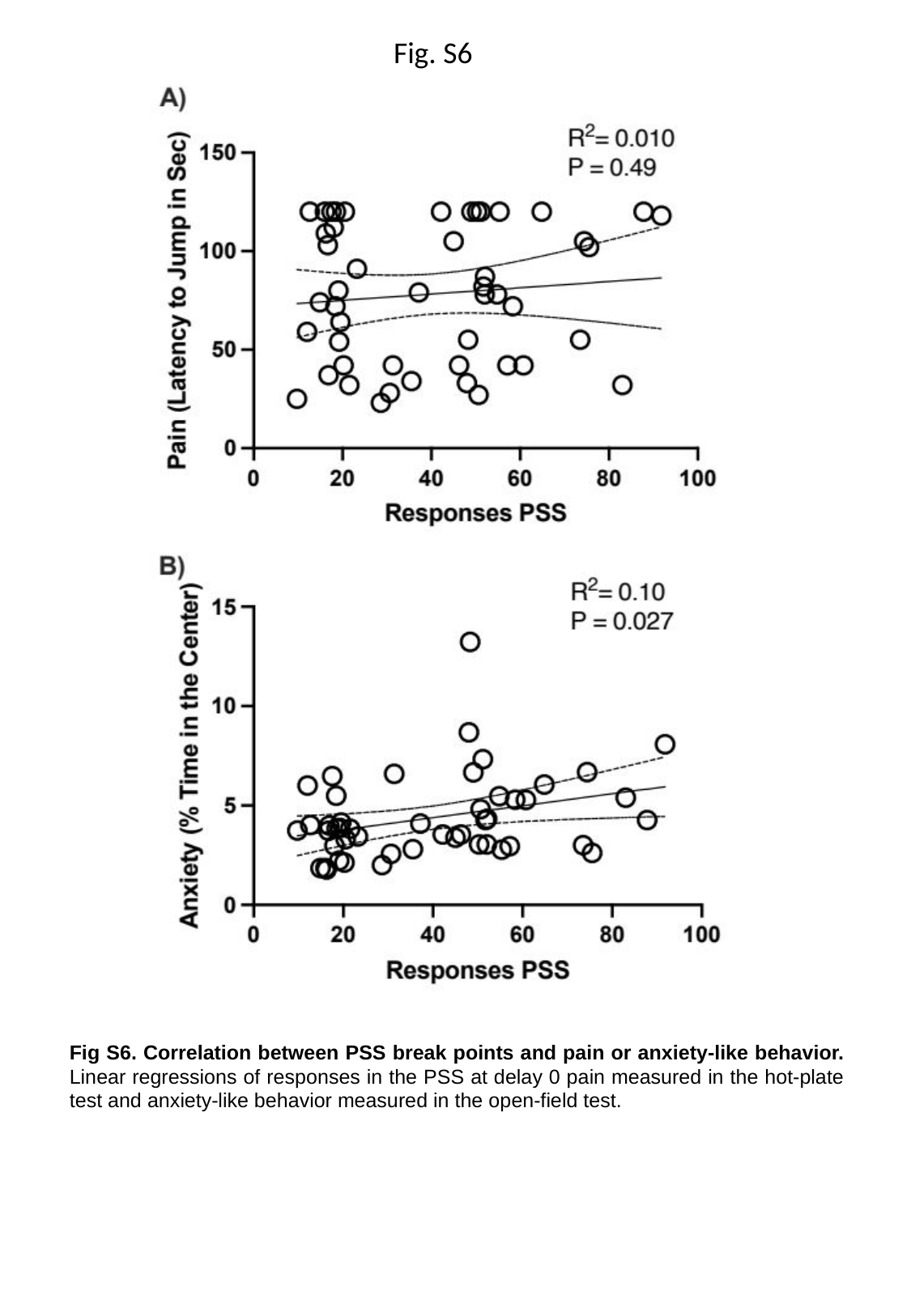

Fig. S6
Fig S6. Correlation between PSS break points and pain or anxiety-like behavior. Linear regressions of responses in the PSS at delay 0 pain measured in the hot-plate test and anxiety-like behavior measured in the open-field test.
